# Altered Levels of Decidual Immune Cell Subsets in Fetal Growth Restriction, Stillbirth, and Placental Pathology

**DOI:** 10.3389/fimmu.2020.01898

**Published:** 2020-08-20

**Authors:** Romy E. Bezemer, Mirthe H. Schoots, Albertus Timmer, Sicco A. Scherjon, Jan Jaap H. M. Erwich, Harry van Goor, Sanne J. Gordijn, Jelmer R. Prins

**Affiliations:** ^1^Department of Obstetrics and Gynecology, University Medical Center Groningen, University of Groningen, Groningen, Netherlands; ^2^Division of Pathology, Department of Pathology and Medical Biology, University Medical Center Groningen, University of Groningen, Groningen, Netherlands

**Keywords:** pregnancy, macrophage, regulatory T cell, natural killer cell, placental pathology, fetal growth restriction, stillbirth

## Abstract

Immune cells are critically involved in placental development and functioning, and inadequate regulation of the maternal immune system is associated with placental pathology and pregnancy complications. This study aimed to explore numbers of decidual immune cells in pregnancies complicated with fetal growth restriction (FGR) and stillbirth (SB), and in placentas with histopathological lesions: maternal vascular malperfusion (MVM), fetal vascular malperfusion (FVM), delayed villous maturation (DVM), chorioamnionitis (CA), and villitis of unknown etiology (VUE). Placental tissue from FGR (*n* = 250), SB (*n* = 64), and healthy pregnancies (*n* = 42) was included. Histopathological lesions were classified according to criteria developed by the Amsterdam Placental Workshop Group. Tissue slides were stained for CD68 (macrophages), CD206 (M2-like macrophages), CD3 (T cells), FOXP3 [regulatory T (Treg) cells], and CD56 [natural killer (NK) cells]. Cell numbers were analyzed in the decidua basalis using computerized morphometry. The Mann-Whitney *U*-test and Kruskal Wallis test with the Dunn's as *post-hoc* test were used for statistical analysis. Numbers of CD68^+^ macrophages were higher in FGR compared to healthy pregnancies (*p* < 0.001), accompanied by lower CD206^+^/CD68^+^ ratios (*p* < 0.01). In addition, in FGR higher numbers of FOXP3^+^ Treg cells were seen (*p* < 0.01) with elevated FOXP3^+^/CD3^+^ ratios (*p* < 0.01). Similarly, in SB elevated FOXP3^+^ Treg cells were found (*p* < 0.05) with a higher FOXP3+/CD3+ ratio (*p* < 0.01). Furthermore, a trend toward higher numbers of CD68^+^ macrophages was found (*p* < 0.1) in SB. Numbers of CD3^+^ and FOXP3^+^ cells were higher in placentas with VUE compared to placentas without lesions (*p* < 0.01 and *p* < 0.001), accompanied by higher FOXP3^+^/CD3^+^ ratios (*p* < 0.01). Elevated numbers of macrophages with a lower M2/total macrophage ratio in FGR suggest a role for a macrophage surplus in its pathogenesis and could specifically indicate involvement of inflammatory macrophages. Higher numbers of FOXP3^+^ Treg cells with higher Treg/total T cell ratios in VUE may be associated with impaired maternal-fetal tolerance and a compensatory response of Treg cells. The abundant presence of placental lesions in the FGR and SB cohorts might explain the increase of Treg/total T cell ratios in these groups. More functionality studies of the observed altered immune cell subsets are needed.

## Introduction

Five to ten percent of pregnancies is complicated by fetal growth restriction (FGR) (1). FGR is defined as a fetus that is unable to reach its intrinsic growth potential (2). It is associated with an increased risk of neonatal mortality and morbidity (3, 4) and adverse long term effects, like impaired neurodevelopment and cardiovascular, endocrine, and metabolic diseases (5). Abnormal placentation leading to placental insufficiency is the most common underlying pathophysiology in FGR (6–8). Additionally, placental senescence is seen with upregulation of pro-inflammatory cytokines, contributing to placental inflammation and oxidative stress (8). FGR is thought to be responsible for nearly one third of stillborn (SB) infants (1, 5), however, a large part of intrauterine deaths remains unexplained (9). Numbers of SB are large, with an estimated 2.6 million cases in 2015 worldwide (10). Placental pathology is a common finding in SB and FGR (11, 12). The main placental lesions found in pregnancy complications are classified as maternal vascular malperfusion (MVM), fetal vascular malperfusion (FVM), delayed villous maturation (DVM), chorioamnionitis (CA), and villitis of unknown etiology (VUE) (13).

The role of the maternal immune system in pregnancy complications has gained increasing attention, with specific interest for the involvement of immune cell subsets in placental development and functioning, and maternal-fetal tolerance. The maternal immune system is subjected to paternal antigens expressed by the fetus via direct contact between fetal trophoblast cells, present in the villous tissue and spiral arteries, and maternal blood (14). Moreover, transfer of fetal and/or placental cells and DNA through the placental barrier into the maternal circulation occurs throughout gestation, referred to as micro-chimerism (15–18). This requires a state in which maternal immune cell subsets cooperate to ensure a tolerogenic environment for the developing fetus (19).

In healthy pregnancies, immune cells are balanced toward an immunoregulatory phenotype. For example, macrophages predominantly express an anti-inflammatory, M2-like phenotype that is involved in tissue remodeling and homeostasis (20–24), numbers of Treg cells participate in preserving maternal-fetal tolerance (25–31), and numbers increase in response to fetal alloantigen (32), and NK cells show a reduction of the cytotoxic subset and an increase in NK cells with immunomodulatory potential (33–36). Furthermore, macrophages and NK cells have been attributed important roles in various aspects of placental development, including trophoblast invasion, spiral artery (SA) remodeling, and tissue regeneration and angiogenesis (37–43). Moreover, exaggerated maternal immune responses can be seen in combination with inflammatory histologically placental lesions that are commonly found in pregnancy complications (44, 45). Inadequate adaptation of the maternal immune system, in particular at the maternal fetal interface, has been widely described in adverse pregnancy outcomes like preeclampsia (29, 46–49), preterm birth (50–52), and spontaneous pregnancy loss (28, 53, 54). However, little is known about possible immune cell imbalances in FGR and SB. As placental malfunctioning is thought to be a key factor in the cause or exacerbation of these complications of pregnancy (6–8, 11, 12), it is likely that altered immune cell balances are at play.

Obtaining more insight into the presence of decidual macrophages, T cells, Treg cells, and NK cells in healthy and complicated pregnancies provides a basis for better understanding the role of these immune cells in the pathogenesis of pregnancy complications. The present study therefore aims to explore the number of decidual macrophages, the M2 macrophage subset, T cells, Treg cells, and NK cells in FGR and SB, and associated types of placental lesions, MVM, FVM, DVM, CA, and VUE, in order to generate hypotheses on immune cell subsets involved in these pregnancy complications.

## Materials and Methods

### Study Design

Study cases with placenta samples and histology reports of three historical study cohorts were included. The DIGITAT cohort (Disproportionate Intrauterine Growth Intervention At Term) included women between 36 and 41 weeks of pregnancy with a singleton with suspected FGR diagnosed by a small for gestational age (SGA) fetus: a fetal abdominal circumference below the 10th centile, estimated fetal weight below the 10th centile or a decreased relative growth in the third trimester (as judged by a clinician) though still above the 10th centile. Exclusion criteria were a history of cesarean section (CS), serious congenital defects, ruptured membranes, renal diseases, diabetes mellitus, or a positive HIV serology (55). The ZOBAS cohort (“Zinnig Onderzoek Bij Antepartum Sterfte,” transl. Useful Examination in Antepartum Death) included all singleton intrauterine fetal deaths diagnosed antepartum after 20 weeks of gestation. SB was diagnosed if fetal heartbeat ceased before labor. We included all women who gave birth from 36 weeks of gestation onwards. Exclusion criteria were pregnancy terminations (for congenital malformations) and intrapartum deaths (56). The NORMA cohort comes from a local study performed at the University Medical Center Groningen that included healthy pregnancies. Fetuses with a birth weight above the 10th centile and below the 90th centile were selected as our control cohort. Women who gave birth from 36 weeks onward were included, provided that their blood pressure was within the normal range, their pregnancies were not complicated by preeclampsia, and women did not use medication. In total, samples of 250 FGR, 64 SB, and 42 healthy pregnancy cases could be included. The hematoxylin eosin (HE) stained tissue slides were re-analyzed and classified for placental histologic lesions by a single perinatal pathologist blinded for clinical details according to the latest international criteria developed by the Amsterdam Placental Workshop Group (13).

### Immunohistochemistry

After delivery, the placentas were fixed in formalin; one full-thickness sample of normal-appearing parenchyma from within the central two-thirds of the placental disc for each case was taken, and embedded in paraffin. Biopsies were taken according to the hospital sampling protocol as later described by the Amsterdam Placental Workshop Group (13). Tissue sections of 3 μm thickness were cut in consecutive slides where possible. Primary antibodies used were CD68 (PGM1, Agilent Dako, USA), CD206 (5C11, BioRad, USA), CD3 (2GV6, Ventana, USA), FOXP3 (236A/E7, Abcam, USA), and CD56 (MRQ-42, Ventana, USA). IHC was performed manually for CD206 and FOXP3. Slides were deparaffinized with xylene and washed in PBS. Antigen retrieval was performed with 10 mM Tris/1 mM EDTA pH 9.0 solution in the microwave for 15 min at 300 or 500 Watt, cooled down for 20 min at room temperature and washed with PBS for 5 min. Blocking endogenous peroxidase was done using 500 μl 30% H_2_O_2_ in 50 ml PBS for 30 min. Slides were washed in PBS for 5 min and incubated for 60 min with the primary antibody FOXP3 in a dilution of 1:100, or with the primary antibody CD206 in a dilution of 1:800. Slides were then again washed in PBS for 5 min, followed by incubation with the peroxidase-labeled rabbit-anti-mouse (RAMpo) secondary antibody (Agilent Dako, USA) with 1% NHS in a dilution of 1:100 for 30 min and the tertiary peroxidase-labeled goat-anti-rabbit (GARpo) antibody (Agilent Dako, USA) with 1% NHS diluted 1:100 for 30 min with an additional washing step after each antibody incubation. Slides were incubated for 10 min in ultra 3,3′-diaminobenzidine-tetrahydrochloride (DAB) peroxidase, diluted in 50 ml PBS and 50 μl 30% H_2_O_2._ After washing in demi water, hematoxylin counterstaining was performed. Slides were dehydrated, dried and covered with mounting medium and a cover slip. For CD68, CD3, and CD56, IHC was performed with the Ventana Benchmark Ultra machine with the Ultraview DAB Kit (Ventana, USA). Deparaffinizing the slides and blocking of endogenous peroxidase is an automatized process performed in the machine at 72°. Antigen retrieval was performed with a 10 mM Tris/1 mM EDTA pH 9.0 solution 52 min for CD68 and 36 min for CD3 and CD56. Slides were incubated with the primary antibodies, 28 min for CD68 and 32 min for CD3 and CD56. CD68 was diluted to 1:100, CD3 and CD56 antibodies were delivered ready to use. For CD56, an Amplification Kit (Ventana, USA) was used to increase the signal intensity of the CD56 primary antibody. The Tissue-Tek Prisma E2S machine was used to automatically perform the DAB reaction, hematoxylin counterstaining, and dehydration of the slides and application of a cover slip.

Slides were scanned with the Philips Intellisite Pathology Solution Ultra-Fast scanner 1.6.1.1.12. In the scans for FOXP3, the number of cells was manually counted due to the low number of cells in each tissue slide. Digital analysis was performed using Visiopharm version 2018.4. In order to create automatic detection classifiers to count the number of cells, scans for CD68, CD206, CD3, and CD56 were analyzed with QuPath version 0.1.2 (57). In short, distinct cell types (e.g., stromal cells, immune cells, background staining) were manually labeled in decidual tissue based on staining pattern and cell morphology. With this labeling set as example, an automatic detection classifier was built and applied on all tissue slides to count the immune cells of interest. Separate classifiers were developed for each immune cell subset. Detailed instructions can be found online (58).

An example of the detection classifier developed to analyze CD68^+^ macrophages can be found in Supplementary Figure 1. In both programs, all decidua basalis tissue in the tissue section was encircled as region of interest. Availability of viable decidual tissue, therefore size of the encircled area, varied per section. Placental septa, decidua tissue overlying large villous infarcts, areas consisting of fibrin without visible trophoblast cells and decidualized endometrium stromal cells and areas with hemorrhage were not included in the encircled surface area. The encircling of the decidua and the development of the detection classifier was performed by two of the authors. As shown in Supplementary Figure 1, the detection classifier distinguished three levels of color intensity for each IHC stain. All positive stained cells were analyzed as immune cells, regardless of the detected intensity of staining. The decidual surface was given in μm^2^ and the outcome calculated as number of cells/mm^2^ decidual tissue.

### Statistical Analysis

IBM SPSS Statistics 23 was used for statistical analyses. QQ-plots and the Shapiro-Wilk test were used to test the normality of the distribution. The Chi-square test was used for categorical data, the independent sample *T*-test for normally distributed continuous data and the Mann-Whitney *U*-test for not normally distributed continuous data. To compare between multiple groups, the Chi-square test was used for categorical data with pairwise Chi-square with Bonferroni correction as a *post-hoc* test. The one-way ANOVA was used for normally distributed data with Tukey's HSD as a *post-hoc* test. The Kruskal Wallis test was used for not normally distributed data with the Dunn's non-parametric comparison as a *post-hoc* test. Similarity of distributions across the patient groups was confirmed by visual inspection of boxplots. Finally, to determine whether the associations between immune cell subsets and patient groups (FGR, SB, healthy controls, and placental lesion groups) were independent of smoking status, we performed linear regression analyses. A *p* < 0.05 was considered significant, a *p* < 0.1 was considered a statistical trend.

### Ethics

Approval of the Medical Ethical Evaluation Committee (METc) has been obtained for the DIGITAT (Leiden University medical Center, Leiden, the Netherlands: P04.210), and ZOBAS (University Medical Center Groningen, Groningen, the Netherlands: M02.00671). The present study has been conducted in accordance with the METc recommendations. For the NORMA study placental tissue was used according to the code of conduct for responsible use following the guideline from the Federation of Medical Scientific Associations with approval of the METc.

## Results

Patient characteristics are presented in Table 1. No significant differences were found for maternal age, fetal sex, and parity between FGR and SB and healthy pregnancies. Gestational age (GA) at birth, birth weight, and placental weight were lower in the FGR group (*p* < 0.001, *p* < 0.001, and *p* < 0.001, respectively) and SB group (*p* < 0.001, *p* < 0.001, and *p* < 0.001, respectively) compared to the control group. More neonates from the FGR and SB groups had a birth weight <p3 compared to the control group (*p* < 0.001 and *p* < 0.001, respectively). The percentage of women who smoke during pregnancy was higher in the FGR group compared to control group (*p* < 0.05).

**Table 1:** Patient characteristics of the FGR, SB, and control cohort.

	**Control (*n* = 42)**	**FGR (*n* = 250)**	**SB (*n* = 64)**
Maternal age (years)	31.51 ± 4.83	29.39 ± 5.50	31.66 ± 4.84
Maternal BMI (kg/m^2^)	–	23.66 ± 5.30	27.12 ± 5.50
Smoking during pregnancy	10 (23.8%)	100 (40%)^*^	18 (28.1%)
**Parity**			
Nulli (0)	19 (45.2%)	152 (60.8%)	29 (43.8%)
Primi (1)	15 (35.7%)	60 (24%)	13 (20.3%)
Multi (2–4)	5 (19.0%)	35 (14%)	19 (28.1%)
Grande multi (>4)	0 (0%)	3 (1.2%)	3 (4.7%)
GA at birth (days)	278.45 ± 11.98	270.45 ± 9.83^***^	275.33 ± 11.32^***^
**Sex**			
Boy	23 (54.8%)	101 (40.4%)	32 (50%)
Girl	19 (45.2%)	147 (58.8%)	30 (46.9%)
Neonatal weight (grams)	3464.88 ±457.48	2296.42 ± 340.52^***^	3169.52 ± 639.821^***^
**Neonatal weight centile**			
<p3	0 (0%)	203 (81.2%)^***^	12 (18.8%)^***^
p3–p5	0 (0%)	16 (6.4%)	6 (9.4%)
p5–p10	0 (0%)	15 (6.0%)	4 (6.3%)
>p10	42 (100%)	14 (5.6%)	40 (62.6%)
Placental weight (grams)	537.00 ± 97.14	360.71 ± 86,46^***^	436.18 ± 113.49^***^

*Patient characteristics are shown for the FGR (n = 250), SB (n = 64), and control (n = 42) cohorts. Continuous data is presented as mean + SD and compared between groups using the one-way ANOVA with Tukey HSD as a post-hoc test. Categorical data is presented as number + percentage and compared using the Chi-square test with Bonferroni correction as a post-hoc test. Significance presented as p-value compared to the control group*.

* *Significant at p < 0.05*.

*** *Significant at p < 0.001. FGR, fetal growth restriction; SB, stillbirth; GA, gestational age*.

### Immune Cell Subsets in Adverse Pregnancy Outcomes

For each immune cell, we determined the number of cells per mm^2^ decidual tissue and compared this between FGR and healthy pregnancies, and SB and healthy pregnancies. In addition, we determined the CD206^+^/CD68^+^ and FOXP3^+^/CD3^+^ ratios.

#### Immune Cell Subsets in the FGR Cohort: Higher Numbers of Macrophages With Lower M2/Total Macrophage Ratios, and Higher FOXP3^+^/CD3^+^ Ratios

**Figure 1:**
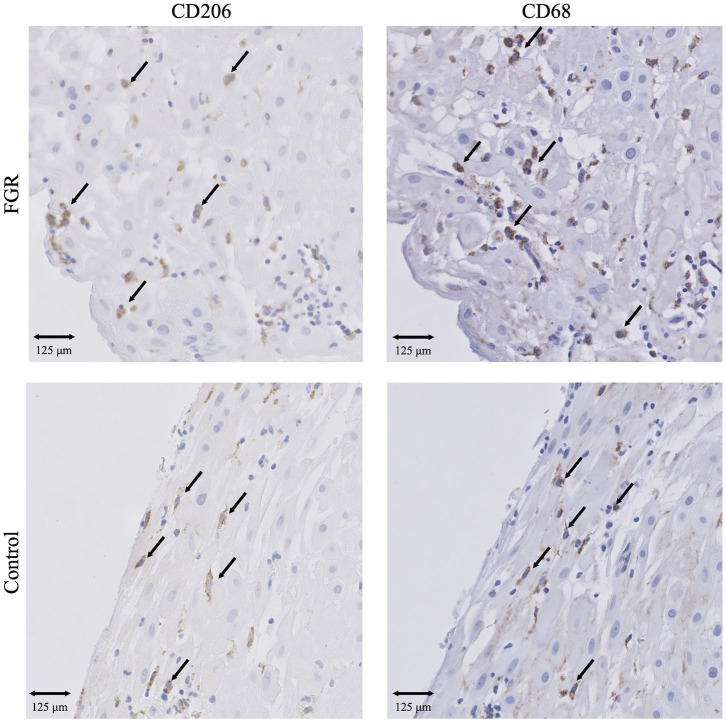
IHC results of macrophages in FGR and controls. Presence of CD68^+^ macrophages and CD206^+^ M2 macrophages in FGR (*n* = 250) and controls (*n* = 42). 20x magnification. Arrows set as example of CD68^+^ and CD206^+^ cells, not all positive cells are indicated. FGR, fetal growth restriction.

**Figure 2:**
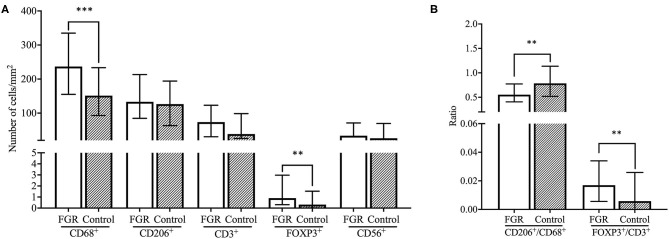
Comparison of immune cell subsets and ratios between FGR and controls. **(A)** Number of cells/mm^2^ are shown for CD68^+^ macrophages, CD206^+^ M2 macrophages, CD3^+^ T cells, FOXP3^+^ Treg cells, and CD56^+^ NK cells and compared between the FGR (*n* = 250) and controls (*n* = 42) cohorts with the Mann-Whitney *U*-test. Data presented as median + IQR. **p < 0.01, ***p < 0.001. **(B)** Ratios of number of cells/mm^2^ are shown for CD206^+^/CD68^+^ macrophages and FOXP3^+^/CD3^+^ T cells and compared between FGR (*n* = 250) and controls (*n* = 42) with the Mann-Whitney *U*-test. Data presented as median + IQR. **p < 0.01. FGR, fetal growth restriction.

The number of CD68^+^ macrophages was significantly higher in decidual tissue of FGR pregnancies compared to control pregnancies (*p* < 0.001) and showed an increase of more than 50% in the FGR group. Although absolute numbers of CD206^+^ cells (M2-like macrophages) were comparable between the FGR pregnancies and control pregnancies, the CD206^+^/CD68^+^ ratio was significantly lower in the FGR cohort compared to controls (*p* < 0.01). In addition, numbers of FOXP3^+^ Treg cells were increased in FGR (*p* < 0.01) accompanied by a higher FOXP3+/CD3+ T cell ratio (*p* < 0.01; Figures 1, 2A,B). Linear regression analysis and comparing the cohorts with exclusion of all women who smoke in the FGR and control cohort, showed that these findings were independent of smoking status. Numbers of CD3^+^ T cells, and CD56^+^ NK cells were comparable between FGR and healthy pregnancies (Figure 2A).

#### Immune Cell Subsets in the SB Cohort: Higher FOXP3^+^/CD3^+^ Ratios

**Figure 3:**
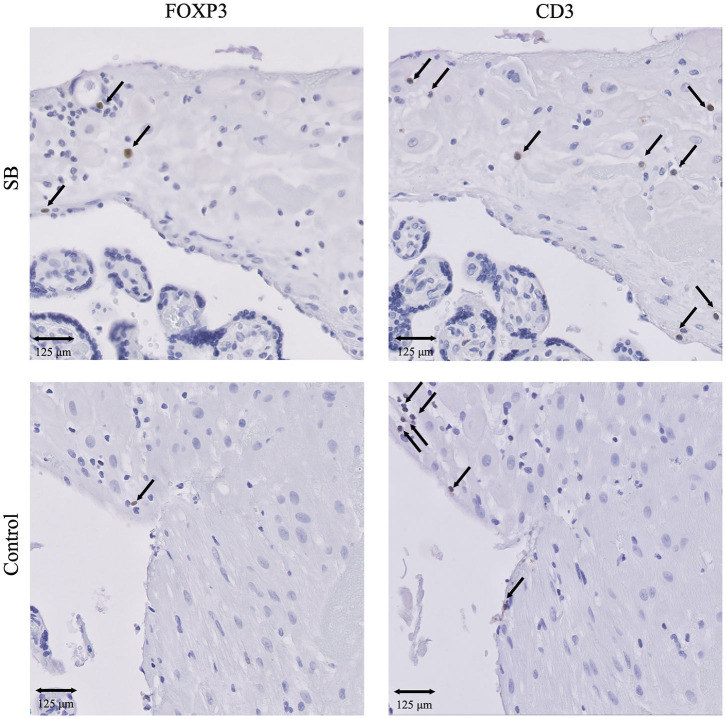
IHC results of T cells and Treg cells in SB and controls. Presence of CD3^+^ T cells and FOXP3^+^ Treg cells in SB (*n* = 64) and controls (*n* = 42). 20x magnification. Arrows indicate all FOXP3^+^ cells. Arrows set as example for CD3^+^ T cells, not all positive cells are indicated. SB, stillbirth.

**Figure 4:**
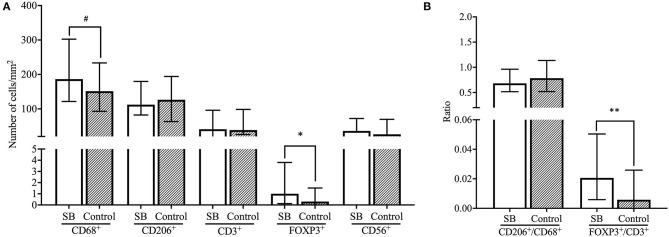
Comparison of immune cell subsets and ratios between SB and controls. **(A)** Number of cells/mm^2^ are shown for CD68^+^ macrophages, CD206^+^ M2 macrophages, CD3^+^ T cells, FOXP3^+^ Treg cells and CD56^+^ NK cells and compared between the SB (*n* = 64) and controls (*n* = 42) cohorts with the Mann-Whitney *U*-test. Data presented as median + IQR. ^#^*p* < 0.1, *p < 0.05. **(B)** Ratios of number of cells/mm^2^ are shown for CD206^+^/CD68^+^ macrophages and FOXP3^+^/CD3^+^ T cells and compared between SB (*n* = 64) and controls (*n* = 42) with the Mann-Whitney *U*-test. Data presented as median + IQR. **p < 0.01. SB, stillbirth.

Significantly higher numbers of FOXP3^+^ T cells were found in SB compared to healthy pregnancies (*p* < 0.05), as well as a higher FOXP3^+^ Treg/CD3^+^ ratio in SB compared to control (*p* < 0.01). These findings were independent of smoking status in the linear regression analysis, however, a significant difference was not found after excluding all women that smoke from the cohorts (Figures 3, 4A,B). Moreover, a trend was found for higher numbers of CD68^+^ macrophages in SB compared to healthy pregnancies (*p* < 0.1), however, the higher numbers of CD68^+^ macrophages did not remain to be a trend after correcting for smoking status. Numbers of CD56^+^ NK cells and CD206^+^ M2-like macrophages were comparable in the SB and healthy pregnancies (Figure 4A). Likewise, no differences in CD206^+^ M2-like/CD68^+^ total macrophage ratio were found in SB compared to healthy pregnancies (Figure 4B).

### Immune Cell Subsets in Placental Lesions

In addition to adverse pregnancy outcomes, we studied the numbers of immune cells in histopathologic placenta lesions.

#### Distribution of Placental Lesions in FGR, SB, and Healthy Pregnancies

**Figure 5:**
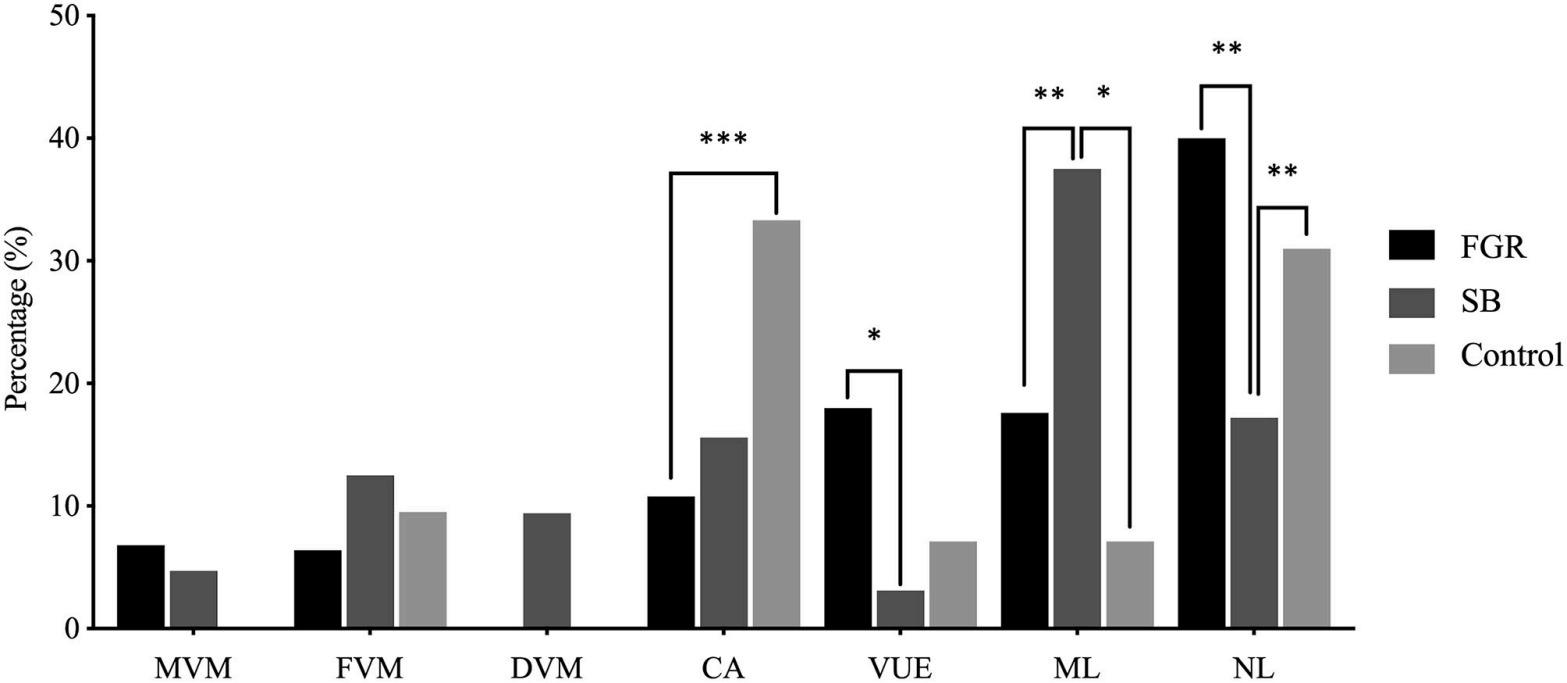
Distribution of placental lesions across the FGR, SB and control cohorts. Percentages of specific lesions, and comparison between the FGR (*n* = 250), SB (*n* = 64), and control (*n* = 42) cohort with the Chi-square test. No cases of DVM as a single lesion were present in the FGR and control cohorts. No cases of MVM as a single lesion were present in the control cohort. *p < 0.05, **p < 0.01, ***p < 0.001 (*p*-values adjusted using Bonferroni correction).

First, the prevalence of lesions was determined in each cohort (Figure 5). Placental lesions were grouped into placentas diagnosed with a single lesion (MVM, FVM, DVM, CA, or VUE), a combination of multiple lesions (ML) and no lesions (NL). Cases with DVM were only observed in the SB cohort. The incidence of multiple lesions was highest in pregnancies complicated with SB compared to FGR (*p* < 0.01) and the control group (*p* < 0.05). In the FGR cohort, the multiple lesion group was predominated by VUE which was present in 45 out of 70 multiple lesions cases (64%). In the SB cohort, the multiple lesion group was predominated by CA present in 19 out of 24 multiple lesion cases (79%). The control cohort had only 4 cases of multiple lesions, predominated by VUE in 3 out of 4 cases (75%). Placentas without lesions were more frequently found in the FGR and control cohort, compared to the SB cohort (*p* < 0.01) and (*p* < 0.01). VUE occurred more in the FGR compared to SB cohort (*p* < 0.05), whereas placentas showing signs of CA were mostly found in the control cohort and showed a significant difference compared to the FGR cohort (*p* < 0.001).

#### Immune Cell Subsets in Placental Lesions in the FGR Cohort: Higher Numbers of T and Treg Cells and Higher Treg/T Cell Ratios in VUE

**Figure 6:**
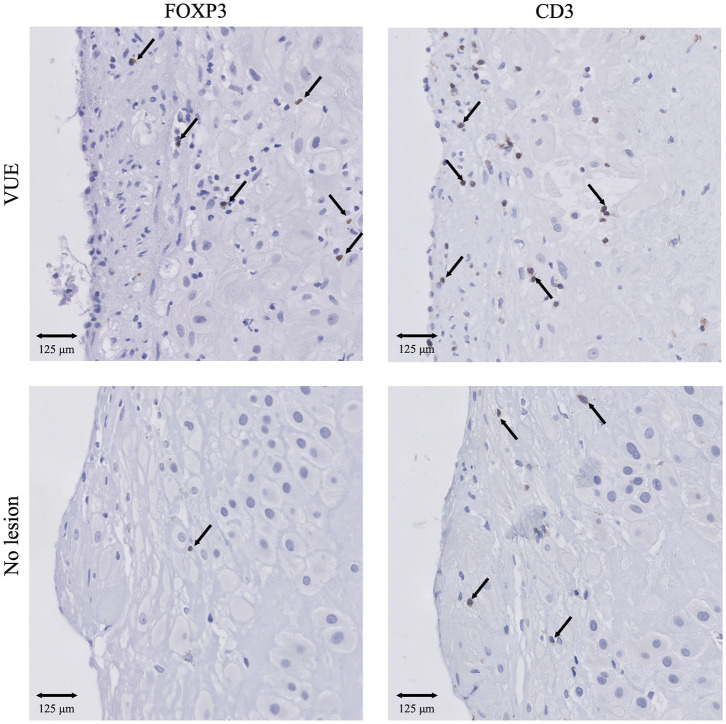
IHC results of Treg cells and T cells in VUE and no lesions in the FGR cohort. Presence of CD3^+^ T cells and FOXP3^+^ Treg cells in VUE (*n* = 45) and no lesion (*n* = 44) within the FGR cohort. 20x magnification. Arrows indicate all FOXP3^+^ cells. Arrows set as example for CD3^+^ T cells, not all positive cells are indicated. FGR, fetal growth restriction; VUE, villitis of unknown etiology.

**Figure 7:**
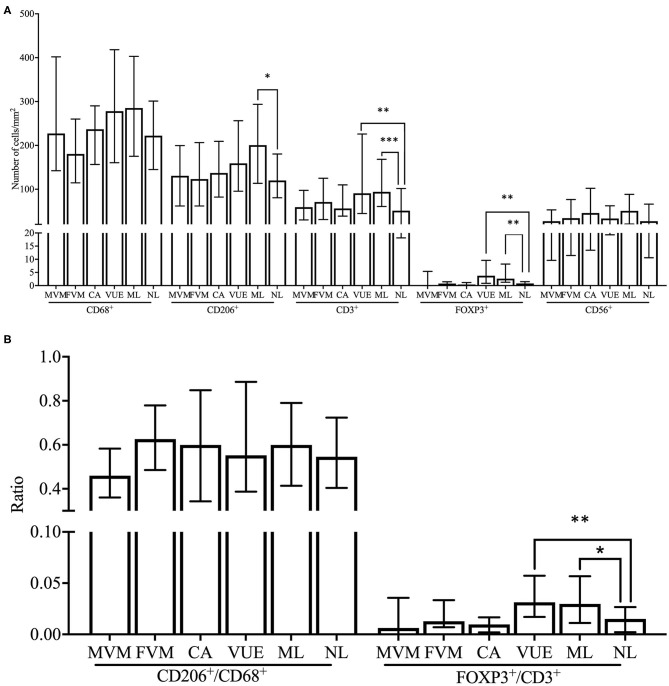
Comparison of immune cell subsets and ratios between placental lesions within the FGR cohort. **(A)** Numbers of cells/mm^2^ are shown for CD68^+^ macrophages, CD206^+^ M2 macrophages, CD3^+^ T cells, FOXP3^+^ Treg cells, and CD56^+^ NK cells and compared between placental lesions [MVM (*n* = 18), FVM (*n* = 17), CA (*n* = 27), VUE (*n* = 45), ML (*n* = 44), and NL (*n* = 44)] within the FGR cohort with the Kruskal Wallis test and pairwise Dunn's non-parametric comparison test as *post-hoc* test. Data presented as median + IQR. *p < 0.05, **p < 0.01, ***p < 0.001. **(B)** Ratios of number of cells/mm^2^ are shown for CD206^+^/CD68^+^ macrophages and FOXP3^+^/CD3^+^ T cells and compared between placental lesions within the FGR cohort with the Kruskal Wallis test and pairwise Dunn's non-parametric comparison test as *post-hoc* test. Data presented as median + IQR. *p < 0.05, **p < 0.01. MVM, maternal vascular malperfusion; FVM, fetal vascular malperfusion; CA, chorioamnionitis; VUE, villitis of unknown etiology; ML, multiple lesions; NL, no lesions.

Due to the low number of cases in each lesion group within the SB and control cohort, we decided to compare the immune cell numbers between placental lesion groups in the FGR cohort only. The number of decidual CD3^+^ T cells was significantly higher in VUE and multiple lesions compared to no placental lesions (*p* < 0.01 and *p* < 0.001, respectively). These higher numbers of CD3^+^ T cells were accompanied by significantly higher numbers of decidual FOXP3^+^ Treg cells in placentas showing signs of VUE (*p* < 0.001) and multiple lesions (*p* < 0.001) compared to placentas without lesions (Figures 6, 7A). To determine if the observed higher numbers of FOXP3^+^ Treg cells were related to the number of CD3^+^ T cells in the decidua, we determined the FOXP3^+^/CD3^+^ ratio. Our results show a significantly higher FOXP3^+^ Treg cell/CD3^+^ total T cell ratio in VUE and the multiple lesion group compared to no lesions (*p* < 0.01 and *p* < 0.05, respectively), which was independent of smoking behavior. This could indicate not only an absolute but also a relative increase in FOXP3^+^ Treg cells (Figure 7B). Higher numbers of CD206^+^ M2-like macrophages were observed in placentas with multiple lesions compared to placentas with no lesions (*p* < 0.05), although this did not result in differences in the CD206^+^ M2-like/CD68^+^ total macrophage ratio (Figures 7A,B). We did not find differences in numbers of CD56^+^ NK cells and CD68^+^ macrophages among the lesion groups.

Since FOXP3^+^/CD3^+^ ratios were increased in both FGR, SB, and the VUE and ML groups, we performed a sub analysis in order to determine whether the presence of VUE and multiple lesions could be responsible for the relative increase of FOXP3^+^ Treg cells in FGR and SB. We excluded all cases of VUE and multiple lesions from the FGR, SB and control cohorts and the differences in FOXP3^+^/CD3^+^ ratios did not remain significant.

## Discussion

The main finding of this study is that in FGR, decidual macrophages appear to have a more inflammatory phenotype compared to healthy pregnancies. Our findings are in accordance with previous studies that found preeclampsia, spontaneous pregnancy loss, and preterm birth to be associated with higher numbers of total macrophages with increased expression of an M1-like, pro-inflammatory macrophage phenotype (47, 50, 53, 54, 59–61). Moreover, a recent study found an increase of placental macrophages in pregnancies in which the fetus showed a decreased growth rate in the third trimester, accompanied by elevated pro-inflammatory markers in the placenta and maternal blood (62). The authors propose placental senescence as a mechanism for placental dysfunction in FGR, underpinned by the absence of elevated CD45^+^ leukocytes and clinical signs of infection, as well as upregulation of DAMPs, associated with oxidative stress, necrosis, and cellular injury. Similar results were seen in a uric-acid induced placental inflammation mouse model, were pups developed FGR and an increase of macrophages was found in the placental junctional zone (63). Contrary to our results, another study has reported lower numbers of CD14^+^ macrophages in the placental bed in FGR compared to healthy pregnancies, although this did not reach a significant level (49). These differences could be due to the use of different markers and/or a different placenta sampling location. To our knowledge, our study is the first to find increases of macrophages in the decidual tissue, accompanied by altered subset balances. Since placental insufficiency in FGR can arise at multiple levels, ranging from early placental developmental defects in trophoblast invasion and SA remodeling, to placental senescence with inflammatory and hypoxic events throughout the course of gestation (8), the question remains from which point in pregnancy the altered macrophage balances originate and how they contribute to the pathogenesis or exacerbation of FGR. Furthermore, the finding that the altered macrophage balances in the decidua appears maternal in origin is an interesting finding in terms of preserving maternal-fetal tolerance and it could be hypothesized that an exaggerated maternal immune response to the fetal and placental tissue is involved. Our results indicate that altered decidual macrophage balances could be involved in FGR and mark the importance of more research on this topic. We suggest performing functionality studies on decidual macrophages and a broader characterization of their phenotype at different time points in gestation to determine their contribution to FGR.

Overall, the incidence of placental lesions was higher in FGR and SB compared to the control group. Interestingly, chorioamnionitis (CA) formed an exception and was found to be more frequently present in placentas from healthy pregnancies, a finding that can most likely be attributed to differences in onset of labor. In the majority of the controls, delivery occurred spontaneously in healthy pregnancies, whereas in the pregnancies complicated by FGR and SB a majority of women had induction of labor. It has earlier been shown that CA is seen more often in pregnancies with spontaneous onset of labor, compared to induction (64) and compared to cesarean section (65). This finding has been explained by the physiological inflammatory process that precedes spontaneous parturition, associated with the presence of inflammatory markers in the myometrium, cervix and chorioamniotic membranes (64). Also in a sub analysis of the DIGITAT trial, CA was more frequently present FGR pregnancies with spontaneous labor compared to induction (66).

When examining the presence of immune cell subsets in common placental lesions within the FGR cohort we showed higher numbers of FOXP3^+^ Treg cells and CD3^+^ T cells, as well as a relative increase in FOXP3^+^ Treg cells in cases of VUE and multiple lesions. VUE is characterized by an inflammatory infiltrate of Hoffbauer cells and maternal CD4+ and CD8+ T cells (67, 68) with irregular involvement of the chorionic villi and areas of placental parenchyma that remain unaffected (69). The etiology of VUE remains poorly understood. Since not an infective organism can be identified, its cause has been explained by either a non-diagnosed underlying infection or an exaggerated maternal immune response to the paternal antigens expressed on the fetal villous tissue (67–70). Interestingly, placentas with VUE show upregulation of MHC class I and II on fetal trophoblast, accompanied by an increase of chemokines within the placenta and maternal and fetal circulation, indicating a systemic inflammatory response in both mother and fetus. This finding supports the hypothesis of a graft vs. host-like response in VUE (71). VUE has been abundantly associated with FGR (69, 70, 72–75). The reported incidence of histological findings of VUE in FGR widely varies, which might be explained by differences in diagnostic criteria. In our study, in which we used the latest international criteria for classifying placental pathology (13), we found VUE to be present in 18% of FGR cases compared to 7% in healthy controls. The increase of Treg cells in VUE at the inflammatory sites in the villous parenchyma, was first shown by Katzman et al. They propose that Tregs infiltrate these sites in order to regulate the immune responses that are potentially harmful toward the fetus (76). As we studied the decidua, we were able to discover higher numbers of Treg cells and T cells in the maternal compartment of the placenta in VUE. We hypothesize that the decidual Treg cells increase in the decidua in order to home toward the villi. The migration of maternal Treg cells could therefore be seen as a mechanism of rescuing the failed maternal-fetal tolerance, which complies with the role of Treg cells in preventing maternal immune responses toward the allogeneic fetus and placenta (27, 28). Earlier, it has been shown that the CD3^+^ T cells infiltrating the fetal villous tissue are indeed maternal in origin (44, 77). We propose similar immunophenotyping and *in situ* hybridization studies with X and Y chromosome probes in both the decidual tissue and placental parenchyma to confirm if this also true for Treg cells.

Like in VUE, we found a relative increase in FOXP3^+^ Treg cells in both FGR and SB, compared to healthy pregnancies. The fact that this not correspond to a reduction in Treg cells that is found in most pregnancy complications, might be attributed to the high number of placental lesions within our study cohorts, since we showed that our results did not remain significant after excluding VUE and multiple lesions in the analysis. Moreover, it could be explained by the fact that we studied the decidua at term. A study in allogeneic mice showed that depletion of Tregs early in pregnancy caused implantation failure and higher resorption rates, while depletion later in pregnancy did not cause pregnancy complications like FGR, hypertension, or proteinuria. It has been suggested that Treg cells are particularly important in the early phase of pregnancy, but not necessarily for the remainder of pregnancy (28), which could explain why we do not find lower FOXP3^+^ expression in our term decidual samples of FGR and SB pregnancies. Notably, numbers of FOXP3^+^ cells are very low in our study cohorts (on average 2–3 cells/mm^2^), likely explained by the physiological decline in Treg cells as delivery approaches (32). We suggest studying Treg cells in the first and second trimester, for example in maternal blood or in placental tissue after pregnancy terminations, to observe possible alterations in Treg cells over the course of FGR and SB pregnancies.

Concerning decidual CD56^+^ NK cells, we did not find differences between FGR and SB and healthy pregnancies. Previously, lower levels of uterine NK cells have been found in FGR (49) and this decline in NK cell levels in FGR has been associated with impaired placental growth and trophoblast invasion (78). Different methodological approaches and inclusion criteria could explain why we could not confirm these changes. Because our detection classifier is finetuned for variations in the intensity of IHC and background stains between tissue slides to prevent major differences in cell count, the three levels of color intensity (as shown for macrophages in Supplementary Figure 1) cannot be used to distinguish CD56^bright^ from CD56^−dim^ subsets. However, since these subsets can, respectively, be attributed cytotoxic and immunoregulatory characteristics (34), it would be interesting to determine if decidual NK subset imbalances might be involved in FGR and SB.

Interestingly, we found an exceptionally high number of women who smoked during pregnancy, 100 out of 250 (40%) in the FGR cohort, 18 out of 64 (28.1%) in the SB cohort, and 10 out of 42 (23.8%) in the control cohort. Smoking is known to be a contributing factor to lower birth weights and to SB (79), and is known to directly harm placental development and vascularization (80). Alterations in immune cell balances in women who smoke during pregnancy (>10 cigarettes per day) have been observed in first trimester decidua and in peripheral blood (81). In order to see whether our results on macrophages and Treg cells in the FGR and SB cohort could be influenced by women's smoking behavior, we performed a linear regression analysis, showing that our results on macrophages and Treg cells in the FGR cohort and Treg cells in the SB cohort were independent of smoking status. Additionally, we conducted a sub-analysis in which we excluded all women who smoked from the three cohorts. Our main finding, the higher numbers of macrophages in FGR with a decrease of the anti-inflammatory subset, again remained significant, indicating a strong association between FGR and altered macrophage levels.

In the selected cases with available histopathologic data from the DIGITAT (FGR) cohort, 203 out of 250 children were born with a birth weight below the third centile (81.2%) compared to the complete DIGITAT study (21.5%). There is a high probability of inclusion bias with more severe cases included in this study, a finding which can be explained by a tendency to only submit the clinically severest cases for placental histopathologic analysis. On the other hand, it is known that focusing on SGA (usually cut-off <p10) in a study cohort of FGR provides an overestimation of the fetuses that are truly growth restricted in that SGA group (82). By using the worst cases (<p3) for our analysis chances are low that the results are diluted by healthy SGA fetuses. However, we strongly advocate to routinely perform histopathologic examination of all suspected FGR pregnancies to gain a better understanding of the pathophysiology and immunology of this complication.

The hypotheses discussed in this study are strengthened by the extensive number of placenta samples that were included from large databases derived from the DIGITAT (FGR), ZOBAS (SB), and NORMA (healthy pregnancies) studies. We have chosen to adopt the predefined inclusion criteria from these studies, except for the GA at birth in order to match pregnancy duration between our cohorts. This could have compromised the use of a narrower definition of FGR, SB, and healthy neonates, however, we feel that this does not outweigh the benefits of analyzing a large study cohort. By developing a detection classifier for each immune cell subset, we have been able to count the number of immune cells per surface area of decidual tissue in a systematic manner reducing observer variability. As the use of historic patient cohorts limits the availability of the tissue samples, a selection of IHC markers had to be made. In order to investigate a variety of immune cells, we have chosen to use one IHC marker for each subset. No double staining was performed for this study. Although these markers were carefully selected, the use of one IHC marker for each subset does limit the reliability of immune cell identification. In the DIGITAT, ZOBAS, and NORMA cohorts, biopsies of the superficial part of the decidua basalis were taken. For future research, it would be interesting to investigate placental bed biopsies in order to determine the localization of immune cells with respect to spiral arteries. It must furthermore be mentioned that, as applies to any IHC study, the observed decidual immune cell populations could be the result of non-specific immune cell infiltration that reflect a maternal immune response to mechanisms like infarction, hypoxia, or inflammation. The tissue was cut in consecutive slides were possible and it can therefore be assumed that numbers of total macrophages and T cells were rather equal so that CD206^+^/CD68^+^ and FOXP3^+^/CD3^+^ ratios could be determined in our samples. Since only paraffin placental tissue was available from our cohorts, no functionality tests could be performed. Even though we consider this to be a limitation of our study, we believe that this methodology does not compromise our results, nor the aim of this study. Our findings bring focus to specific immune cell subsets that are likely involved in the pathogenesis of FGR, SB, and placental lesions and clearly show immune cell imbalances in these pregnancy complications. We urge for more in-depth research with elaborate immunophenotyping and functionality studies to better characterize these immune cell subsets.

## Conclusion

FGR and SB are complex and multifactorial pregnancy complications of which the immunologic background has received little attention. Due to our historical databases consisting of a large number of FGR and SB cases with systematically classified data on placental histopathologic lesions, this study is the first to analyze immune cell subsets in these adverse pregnancy outcomes and placenta lesions on a large scale. Our study indicates a role for macrophages in the pathophysiology of FGR, with an additional relative decrease of an anti-inflammatory macrophage subset in FGR. Additionally, our findings point toward a relative increase of Treg cells in VUE, FGR, and SB, possibly explained by a compensatory response to rescue the failed maternal-fetal tolerance. These insights highlight the importance of further investigating the functional activity and subset balances of macrophages and Treg cells in pregnancies complicated by FGR, SB, and placental lesions.

## Data Availability Statement

The raw data supporting the conclusions of this article will be made available by the authors, without undue reservation.

## Ethics Statement

The studies involving human participants were reviewed and approved by Leiden University Medical Center, Leiden, the Netherlands: P04.210 University Medical Center Groningen, Groningen, the Netherlands: M02.00671. The patients/participants provided their written informed consent to participate in this study.

## Author Contributions

RB designed the study, performed the experiments, analyzed the data, and wrote the manuscript. MS designed the study, performed the experiments, and edited and reviewed the manuscript. AT, SS, and JE designed the study, reviewed, and edited the manuscript. HG, SG, and JP designed the study, reviewed and edited the manuscript, organized financial support, and supervised the project. All authors approved the submitted version of the manuscript.

## Conflict of Interest

The authors declare that the research was conducted in the absence of any commercial or financial relationships that could be construed as a potential conflict of interest.
