# Supplementary figures and images for: Altered Levels of Decidual Immune Cell Subsets in Fetal Growth Restriction, Stillbirth, and Placental Pathology
Source: Front Immunol. 2020 Aug 20;11:1898. doi: 10.3389/fimmu.2020.01898 (PMC7468421; doi:10.3389/fimmu.2020.01898)

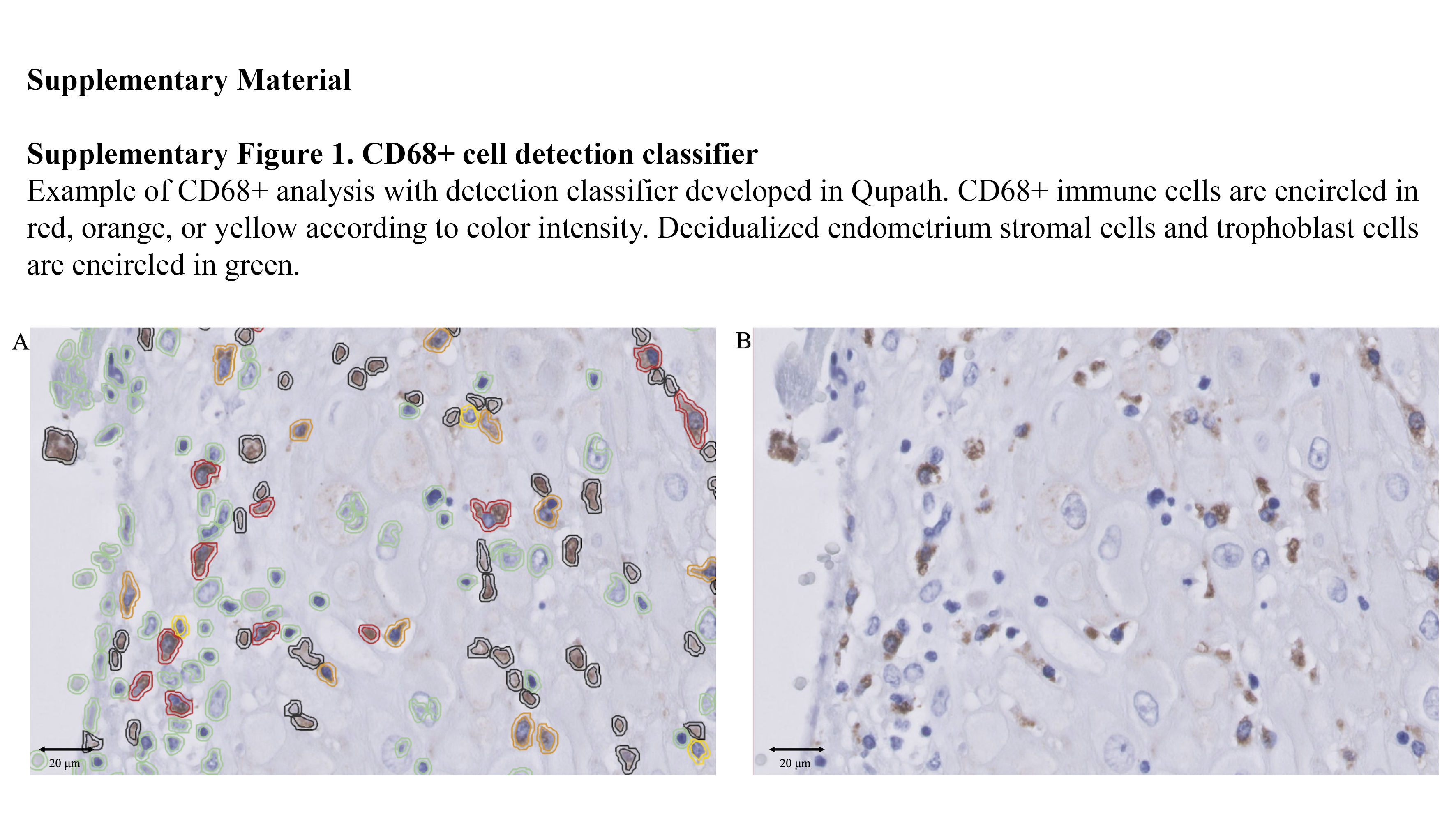

Supplement: Supplementary file 1 [file Image_1.tif]
